# Case report: a case of primary cutaneous diffuse large B-cell lymphoma, leg type with TdT positive in an elderly woman

**DOI:** 10.1186/s13000-026-01768-w

**Published:** 2026-04-01

**Authors:** Jingwei Zheng, Xinghui Li, Hao Chen

**Affiliations:** 1 Hospital for Skin Diseases, Institute of Dermatology, Chinese Academy of Medical Sciences and Peking Union Medical College, Nanjing, 210042 China; 2 Department of Dermatology, Yancheng No.1 People’s Hospital, Affiliated Hospital of Medical School, Nanjing University, The First people’s Hospital of Yancheng, Yancheng, 224005 China; 3 Department of Pathology, Hospital for Skin Diseases, Institute of Dermatology, Chinese Academy of Medical Sciences and Peking Union Medical College, Nanjing, 210042 China

**Keywords:** Primary cutaneous diffuse large b-cell lymphoma, leg type (PCDLBCT-LT), Terminal deoxynucleotidyl transferase (TdT), R-CHOP

## Abstract

**Background:**

Primary cutaneous diffuse large B-cell lymphoma, leg type(PCDLBCL-LT) is a rare form of lymphoma that originates from the post-germinal center and typically does not express terminal deoxynucleotidyl transferase(TdT), a marker of immunophenotypic immaturity.

**Case description:**

A 73-year-old female presented to our hospital with recurrent nodules on her lower limbs, which had persisted for over five years without systemic involvement, accompanied by a gradual increase in TdT expression. Histology revealed diffuse infiltration of centroblasts and/or immunoblasts within the dermis and subcutis, with CD20, CD10, BCL2 and c-MYC expression. Fluorescence in situ hybridization (FISH) showed *MYC* rearrangement without *BCL2*/*BCL6* alterations, corroborating the diagnosis of TdT-positive PCDLBCL-LT. Following six cycle of R-CHOP chemotherapy, the patient achieved complete remission. A follow-up conducted one year showed no tumor recurrence.

**Conclusion:**

Our case showed that TdT can be expressed in PCDLBCL-LT, and highlight the importance of a comprehensive analysis of clinicopathology and ancillary testing to avoid misdiagnosis. Additionally, the clinical course of TdT-positive PCDLBCL-LT seems remain relatively favorable. However, further research is still needed on this relationship.

## Introduction

Primary Cutaneous Large B-cell Lymphoma (PCLBL), a rare type of non-Hodgkin lymphoma, is classified into distinct subtypes: primary cutaneous follicle center lymphoma (PCFCL), primary cutaneous marginal zone lymphoma (PCMZL), primary cutaneous diffuse large B-Cell lymphoma, leg type (PCDLBCL-LT), EB virus-positive mucocutaneous ulcer (EBVMCU) and intravascular large B-cell lymphoma (IVLBCL) [1, 2]. Among these, PCDLBCL-LT typically occurs in individuals with a median age of 76, shows a slight female predominance, and presents clinically as red or purple nodules, plaques, or masses, which may be accompanied by ulcers. Histopathologically, PCDLBCL-LT is characterized by a diffuse dermal infiltrate, composed of a monotonous proliferation of large centroblasts and/or immunoblasts with high mitotic activity [2, 3]. These atypical lymphoid cells usually originate from post-germinal centers and are immunoreactive for BCL2, MUM1, CD79a, immunoglobulin M (IgM), and mostly BCL6 [2, 3]. Mutations in NF-κB-activating genes are observed frequently in *MYD88*, *PIM1*, and *CD79B*, with *MYD88*^*L265P*^ mutations being the most common and detected in about 75% of PCDLBCL-LT cases [2, 4].

TdT is a kind of DNA polymerase that catalyzes DNA synthesis in a template-independent manner [5, 6]. TdT expression in aggressive B-cell lymphomas is associated with *MYC* rearrangements, immunophenotypic immaturity, and poor prognosis. Apart from immature B/T lymphocytes, certain mature B-cell lymphomas may present as mixed phenotypes or rare subtypes with TdT expression [1, 2, 5]. We present an exceedingly rare case of TdT-positive PCDLBCL-LT in a 73-year-old woman to describe the clinical course, histopathology and management, and to improve understanding of this disease.

## Case description

**Fig. 1:**
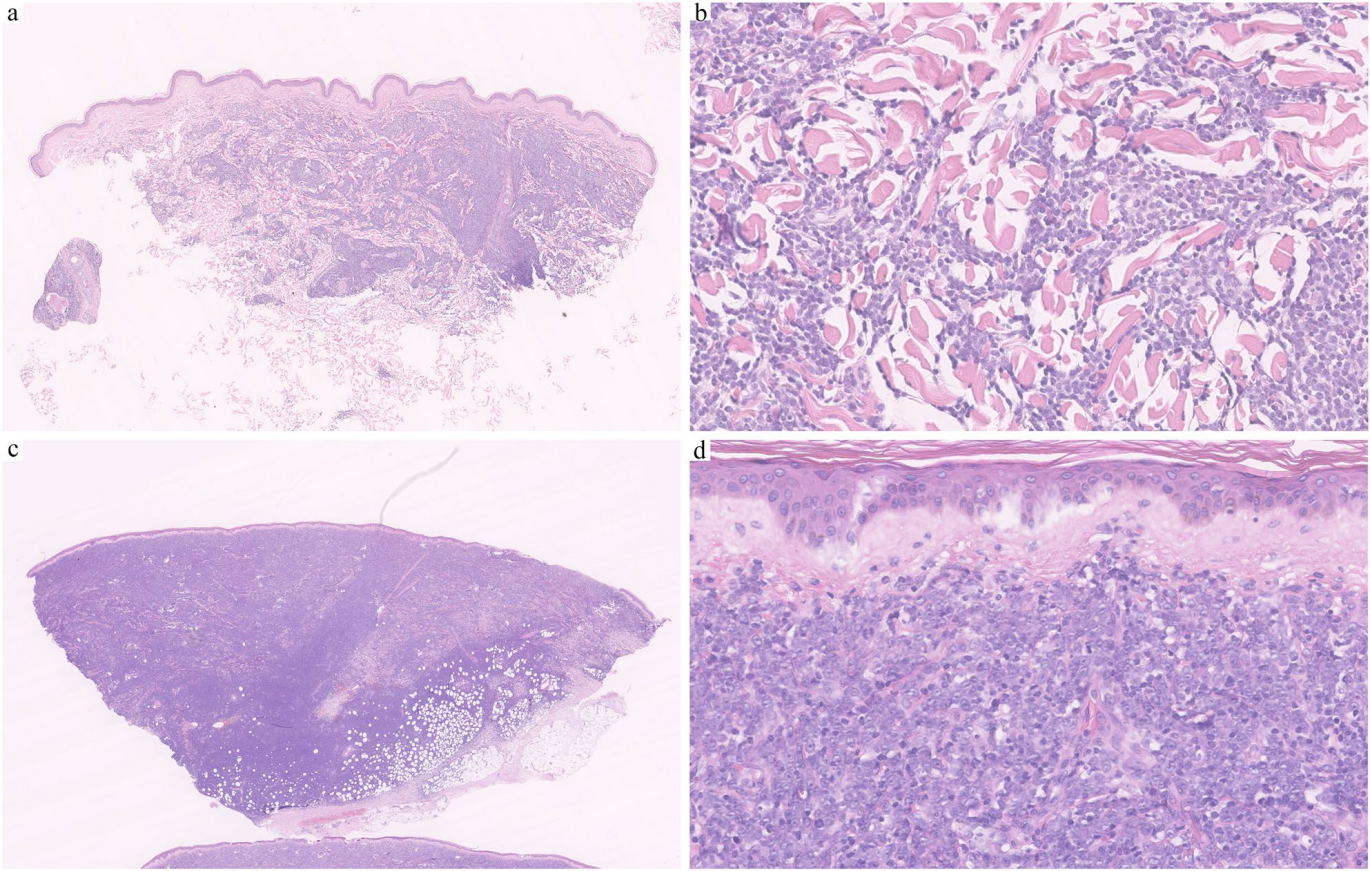
The HE of the patient in 2019 (**a**, **b**) and 2024 (**c**, **d**). **a**, **b** showed nodule or diffuse monotonous immunoblasts infiltrated in the dermis without epidermal involvement, with some arranged in a linear pattern between collagen fibers. **c**, **d** showed diffuse infiltration of centroblasts/immunoblasts in the dermis and subcutis without epidermal involvement

**Fig. 2:**
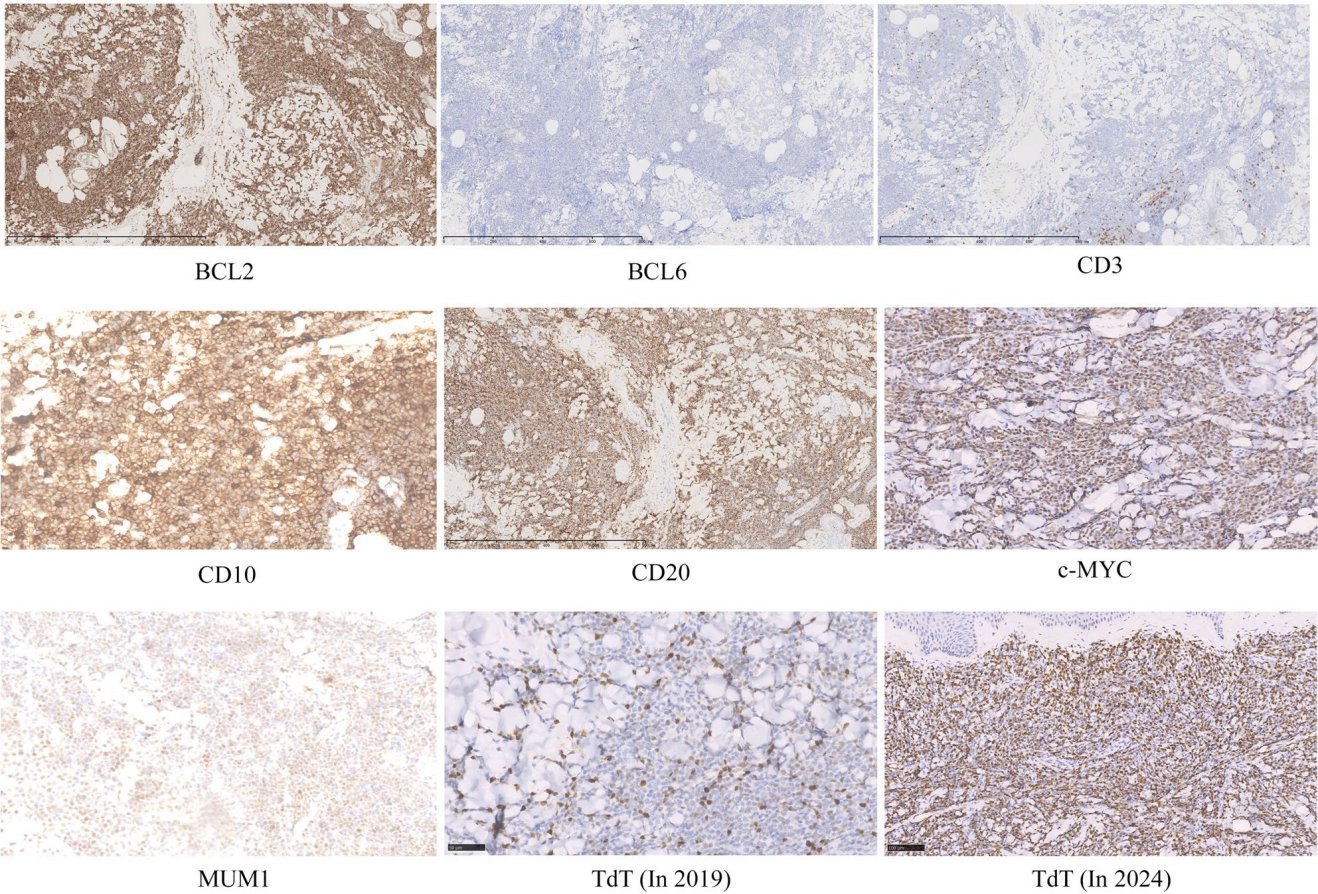
The IHC result of the patient in 2024 showed BCL2, CD10, CD20, c-MYC positive, while BCL6, CD3 and MUM1 negative. TdT expression in 2024 showed over 50% positive

The patient complained of recurrent erythema and nodules on her left chest and legs for five years. The course could date back to November 2019, when the patient first presented with asymptomatic nodules on her left chest wall and legs. The first biopsy revealed dermal diffuse filtration of atypical lymphoid cells (Fig. 1a and b) expressing CD10, CD20, PAX5, BCL2, and c-MYC, while being negative for CD3, BCL6, and MUM1. The biopsy also showed 10% TdT positivity (Fig. 2) and 60% Ki67 positivity. Systemic workup, including blood tests and PET-CT, showed no extracutaneous involvement. We suspected TdT-positive PCDLBCL-LT and recommended hospitalization for further evaluation to exclude conditions such as lymphoblastic lymphoma/leukemia involvement or high-grade lymphoma with TdT positivity. However, the patient declined hospitalization for personal reasons. One month later, the patient’s skin lesions spontaneously resolved.

**Fig. 3:**
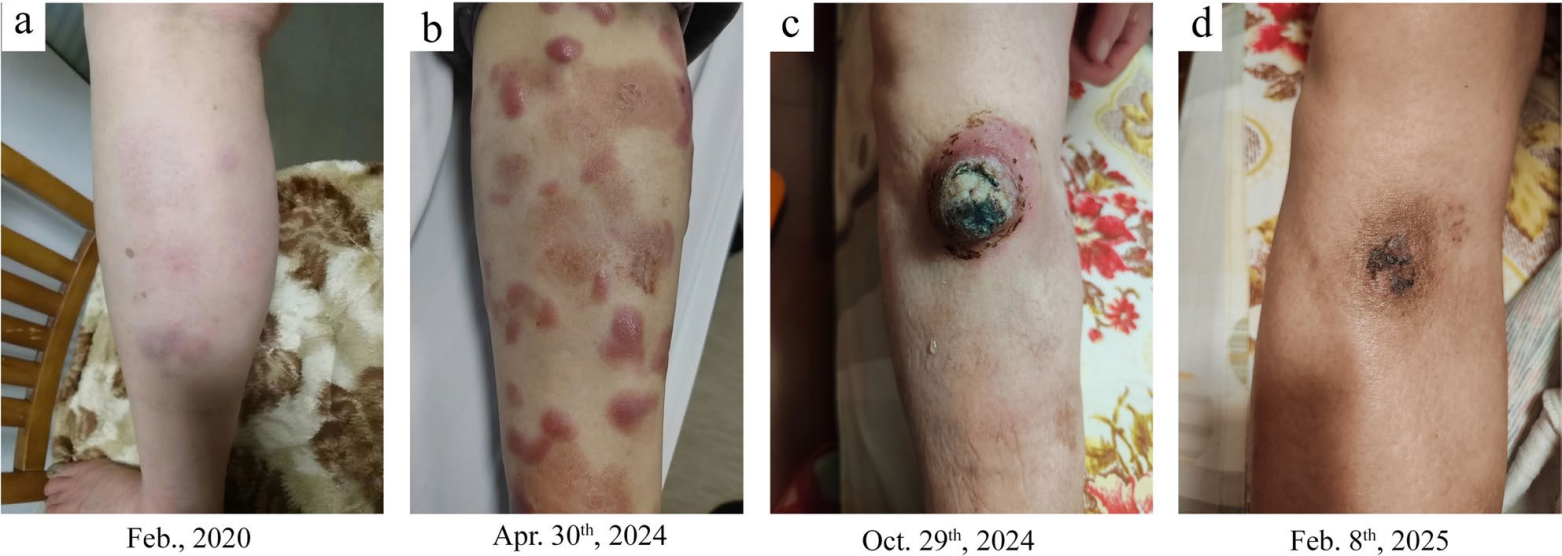
Change in the patient’s skin lesions. **a** Recurrent erythema and erythematous plaques on both lower limbs in February 2020. **b** After taking dexamethasone and thalidomide for nearly two months, the skin lesions partially subsided. **c **A large tumor with ulceration in October 2024. **d** After the final round of chemotherapy, the skin lesions resolved almost completely

By February 2020, the patient’s skin lesions had recurred, presenting as new erythema and erythematous plaques on both lower limbs(Fig. 3a). Despite this, the patient did not seek medical care and instead self-administered traditional Chinese herbal medicine, which lead to complete resolution at one point. From then until 2023, her skin lesions recurred intermittently but improved with the same herbal treatment. Routine examinations during this period revealed no significant abnormalities.

However, after August 2023, the same herbal treatment became ineffective, and erythema and nodules on the lower limbs persisted. In March 2024, the patient returned to our hospital and was again recommended for hospitalization to undergo *BCR* rearrangement testing, systemic evaluations, and cytogenetic studies, but the patient refused. An alternative PET-CT scan revealed multiple subcutaneous lesions in the lower limbs consistent with lymphoma infiltration, and no extra-cutaneous involvement was observed. The patient declined chemotherapy, so dexamethasone and thalidomide therapy was initiated. Subsequently, her lesions improved(Fig. 3b).

**Fig. 4:**
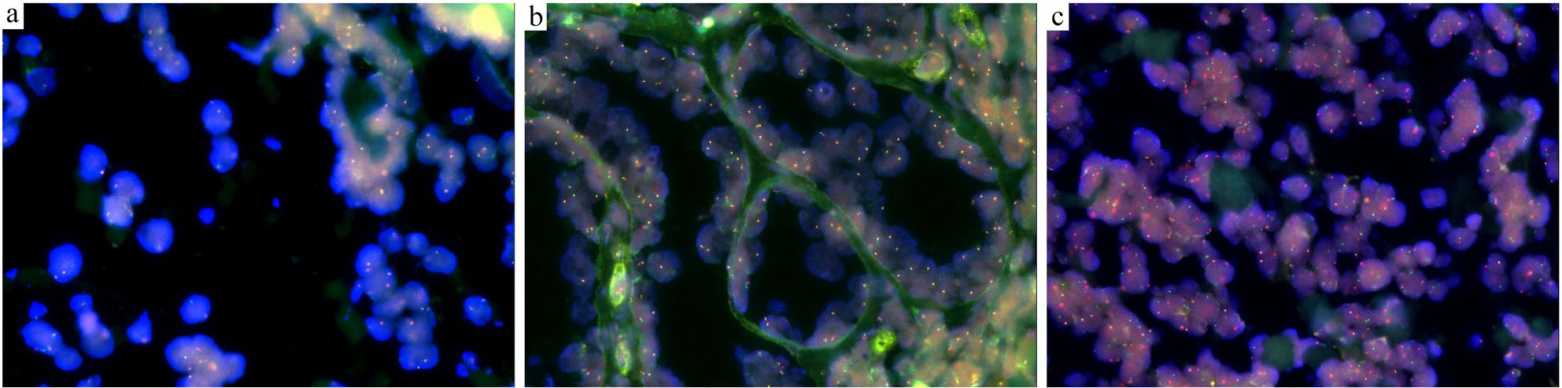
FISH for *BCL2*(**a**), *BCL6*(**b**) and *c-MYC*(**c**) using dual color break apart probe. The upstream probe is labeled with green fluorescence, while the downstream probe is labeled with red fluorescence. The separation of the probes indicates the presence of rearrangement. The rearrangement of c-*MYC* (**c**), but not *BCL2* (**a**) or *BCL6* (**b**), was detected

However, in September 2024, a large nodule relapsed on her right lower leg and progressed to ulceration (Fig. 3c). Routine laboratory tests revealed an increase in monocyte percentage (13%) and lactate dehydrogenase (LDH:301U/L). Flow cytometry for peripheral blood examination was normal. A chest and abdominal CT scan showed multiple enlarged lymph nodes in the right inguinal region, abdomen, and bilateral iliac vessels. A newly conducted biopsy showed diffuse infiltration of centroblasts/immunoblasts in the dermis and subcutis without epidermal involvement. A non-infiltrating zone (Fig. 1c and d) and elevated TdT expression (over 50%) were observed(Fig. 2). Tumor cells expressed CD20, PAX5, CD79a, CD10, BCL2 and c-MYC, but were negative for CD1a, CD3, CD5, CD21, CD23, CD30, CD34, CD117, MUM1, BCL6, ALK, MPO, CD138, CyclinD1 and EBER. FISH analysis showed *MYC* rearrangement without *BCL2*/*BCL6* alterations(Fig. 4). Based on these findings, B lymphoblastic lymphoma(BLL), high-grade B-cell lymphoma(HGBCL), and mixed phenotype acute leukemia (MPAL) were excluded, and the diagnosis of TdT-positive PCDLBCL-LT was confirmed. After persuasion, the patient agreed to undergo R-CHOP chemotherapy, achieving complete remission after six cycles (Fig. 3d). So far, a follow-up conducted one year showed no tumor recurrence.

## Discussion

In B-cell-derived lymphomas, TdT expression can also be observed in immature lymphomas, transformed lymphomas, HGBCL and some exceedingly rare types of other mature lymphomas [5]. In our case, it mainly need to be differentiated from BLL and HGBCL with TdT positivity.

BLL is a malignant tumor derived from immature precursor B lymphocytes, which express markers, such as CD19, CD79a, PAX5, TdT, CD10, and variably CD20 and CD34, but they typically lack mature B-cell markers (e.g., CD23, BCL2, BCL6). Histopathologically, the tumor consists of small to medium-sized round blasts with fine chromatin, inconspicuous or small nucleoli, scant cytoplasm, and frequent mitotic activity (> 90%). BLL predominantly affects children and adolescents, with adult cases being relatively rare. Clinically, it presents either as leukemia (involving bone marrow and peripheral blood) or as lymphoma (localized tumor mass). Common symptoms include anemia, thrombocytopenia, neutropenia, and circulating blasts in the peripheral blood. Extranodal involvement (e.g., skin, bone, or soft tissue) is observed in approximately 50% of BLL cases. However, our patient was an elderly woman whose cutaneous lesions primarily located on the lower legs without systemic involvement, which contrasted with the typical systemic manifestations of BLL. Pathologically, in the second biopsy, the tumor cells showed blastoid feature. Additionally, immunohistochemical staining for BCL2 was positive in this case, another feature not commonly seen in classic BLL, thereby ruling out BLL diagnosis.

TDT positivity is also seen in some HGBCL, a distinct entity from BLL. HGBCL demonstrates higher MYC protein expression (often > 40% positivity) and frequently co-expresses BCL2 and/or BCL6 (the double-hit (DH)/triple-hit (TH) subtypes), with an extremely high ki67 proliferation index (> 90%). The overexpression of BCL2 in HGBCL is typically driven by the t(14;18) translocation. HGBCL progresses extremely rapidly, with tumor doubling within days to weeks and markedly elevated LDH (> 1000 U/L). Patients with HGBCL are more susceptible to hyperuricemia, hyperkalemia, acute kidney injury, and even spontaneous tumor lysis syndrome (TLS). Moreover, HGBCL often demonstrates widespread multi-focal dissemination, with frequent involvement of the liver, spleen, bone marrow (30–50% infiltration), and central nervous system (CNS). Nearly all TdT-positive HGBCL cases exhibit highly aggressive clinical behavior and poor prognosis, even with standard therapy [6–8]. In our case, the patient had recurrent episodes of skin lesions for five years with no systemic involvement and only mild LDH elevation(301U/L). The FISH results showed translocation of *MYC*, but no rearrangement of *BCL2* or *BCL6*. Thus, the diagnosis of HGBCL with TdT positivity was ruled out.

In extremely rare cases, TdT expression has been observed in PCDLBCL-LT, with only two prior reports [9, 10]. Notably, in our case, TdT expression significantly increased in the later biopsy when the patient presented with ulceration. But whether this elevated expression is related to the fluctuation of the disease still requires further study. This heterogeneity has already been reported in the systemic diffuse large B-cell lymphoma(DLBCL), showing that it may reflect the presence of tumor cell subpopulations with distinct proliferation, immune evasion capacity, leading to variations in the distribution and function of immune cells within the tumor microenvironment [5]. However, in our patient, her skin lesions were mainly characterized by recurrent restricted erythema and nodules, without systemic involvement, despite the absence of standard treatment. In the early stage, the lesions even spontaneously resolved, demonstrating an indolent characteristic, suggesting that TdT positivity does not seem to change the relatively favorable clinical course of PCDLBCL-LT. Additionally, *c-MYC* rearrangement has been detected in nearly one-third of PCDLBCL-LTs [11], however, current PCDLBCL-LTs with “immature” characteristics all showed *MYC* rearrangement without accompanying *BCL-2* and *BCL-6* rearrangement, suggesting that this may be a unique variant.

The natural history of PCDLBCL-LT more closely resembles that of systemic DLBCL. Therefore, our patient was effective with the R-CHOP treatment. Relapses/refractory cases are frequent with no standardized therapeutic guidelines. Lenalidomide seems to be an excellent therapeutic option as a second-line treatment of relapsed PCDLBCL-LT [12].

## Conclusions

We report an exceedingly rare case of TdT-positive PCDLBCL-LT, highlighting the importance of comprehensive clinicopathological and molecular analysis. TdT positivity does not appear to alter the relatively favorable clinical course characteristics of PCDLBCL-LT. R-CHOP regimen, with or without radiation therapy, is considered an effective first-line treatment for PCDLBCL, regardless of TdT positivity.

## Data Availability

The raw data supporting the conclusions of this article will be made available by the authors, without undue reservation.
